# Biochemical Characterization and Phylogenetic Analysis of the Virulence Factor Lysine Decarboxylase From *Vibrio vulnificus*

**DOI:** 10.3389/fmicb.2018.03082

**Published:** 2018-12-11

**Authors:** Lifen Han, Jinjin Yuan, Xiulan Ao, Shujin Lin, Xiao Han, Hanhui Ye

**Affiliations:** ^1^The United Innovation of Mengchao Hepatobiliary Technology Key Laboratory of Fujian Province, Mengchao Hepatobiliary Hospital, Fujian Medical University, Fuzhou, China; ^2^Infectious Diseases Hospital of Fuzhou, Fuzhou, China; ^3^College of Biological Science and Engineering, Fuzhou University, Fuzhou, China

**Keywords:** *Vibrio vulnificus*, lysine decarboxylase, virulence factor, phylogenetic analysis, biochemical characterization

## Abstract

Cadaverine is produced in organisms from the amino acid lysine in a decarboxylation reaction catalyzed by lysine decarboxylase (EC 4.1.1.18). The inducible lysine decarboxylase CadA plays a vital role in acid stress response for enteric bacteria. *Vibrio vulnificus* is an extremely virulent human pathogen causing gastroenteritis when the acid conditions that prevent survival of *V. vulnificus* in the stomach or small intestine are overcome. A gene encoding CadA was identified from *V. vulnificus*. Subsequent analyses showed that CadA from *V. vulnificus* (VvCadA) is a decamer with a 82-kDa subunit. Homogenous VvCadA was purified from *Escherichia coli* and used for lysine decarboxylation with an optimal pH of 6.0 and optimal temperature of 37°C. The apparent *V*_max_ and *K*_m_ for lysine were 9.45 ± 0.24 μM/min and 0.45 ± 0.05 mM, respectively. Mutation analysis suggested that the amino-acid-binding pyridoxal phosphate, the cofactor of the enzyme, plays a vital role in the reaction. Mutation of the negatively charged residues interacting with lysine also affected the activity of the enzyme to some extent. Quantitative RT-PCR showed that expression of *VvcadA* was up-regulated under low pH, low salinity, and oxidative stresses. Furthermore, the concentration of cadaverine released to the cell exterior also increased under these stresses. Protein sequence similarity network (SSN) analysis indicated that lysine decarboxylases with ornithine decarboxylases and arginine decarboxylases shared a common ancestor, and that lysine decarboxylases are more conserved during evolution. Our data provide evidence for the biochemical characteristics and important roles of VvCadA under stress conditions.

## Introduction

Of the diverse marine bacteria, *Vibrio vulnificus* is one of the most common causes of gastroenteritis and sepsis (Morris, 1988). Due to high patient mortality during the summer time in Florida, much attention focused on preventing infection by this bacterium (Hlady and Klontz, 1996). Several studies have revealed that factors such as polysaccharide capsules contribute to the disease caused by *V. vulnificus* (Wright et al., 1981; Smith and Merkel, 1982; Simpson and Oliver, 1983; Testa et al., 1984; Kothary and Kreger, 1987; Simpson et al., 1987; Lee et al., 2004b), and motility could be another virulence determinant of this organism (Lee et al., 2004a). However, the major pathogenic determinant is still unclear (Koo et al., 2007). It is well known that for survival in the stomach the organism must evade acidic destruction (Horseman and Surani, 2011). Two different mechanisms are used in this acid tolerance (Jones and Oliver, 2009): up-regulation of lysine decarboxylase and manganese superoxide dismutase (MnSOD) under acid and secondary oxidative stress. Lysine decarboxylase converts L-lysine into cadaverine (1,5-pentanediamine), which is an acid neutralizer (through deamination) and superoxide radical scavenger; MnSOD is also involved in acid neutralization and reduction of oxidative stress (Kim et al., 2005).

There are two kinds of lysine decarboxylases in microorganisms: LdcC and CadA (LdcC). LdcC, a constitutive enzyme encoded by *ldcC*, plays a mainly metabolic role and it is independent of pH changes. The inducible CadA is a key response enzyme for enterobacterial acid stress. CadA, encoded by *cadA* in the *cadBA* operon, converts lysine to carbon dioxide and cadaverine, which is then transported outside the cell by CadB (Merrell and Camilli, 1999; Soksawatmaekhin et al., 2004). Several factors are known to induce *cadBA*, including changes in external pH, lysine, and oxygen. *cadC*, lying upstream from *cadBA*, encodes a membrane-bound protein that has been found to positively regulate expression of *cadBA* (Kim et al., 2006). CadA has been implicated in the adaptive acid tolerance response in *Salmonella enterica*, *Vibrio cholerae*, *V. vulnificus*, and *Escherichia coli* (Kovacikova et al., 2010). When the organisms were exposed to acidic conditions, CadA could quickly provide protection. Furthermore, a *cadA* mutant had decreased tolerance to low pH (Rhee et al., 2002), and mutation in *cadC* also led to low tolerance to low pH, with an accompanying lack of cadaverine (Rhee et al., 2005).

Both lysine decarboxylase (EC 4.1.1.18) and ornithine/arginine decarboxylase are members of the pyridoxal-5′-phosphate (PLP)-dependent enzymes α-family (Eliot and Kirsch, 2004). The structure of CadA from *E. coli* revealed that it forms a decamer by protein oligomer of five dimers (Kanjee et al., 2011a). There are three domains in this protein – an N-terminal wing domain, a PLP-binding core domain, and a C-terminal domain (CTD) (Kandiah et al., 2016). Despite many studies being conducted on the physiological function of CadA in *V. vulnificus* (VvCadA), very little is known about its enzymatic characteristics. Draft or complete genome sequences of several strains of *V. vulnificus* have been reported (Efimov et al., 2015; Chung et al., 2016), and these genomes generally contain one *cadA*, but not *ldcC*.

In the current study, recombinant His-tagged VvCadA was efficiently obtained and purified in a bacterial system. The catalytic characteristics and active sites were further analyzed by point mutagenesis. The expression level of *VvcadA* was also determined under different stress conditions.

## Materials and Methods

### Protein Expression and Purification

*Vibrio vulnificus* was cultivated at 37°C in Luria-Bertani (LB) broth. DNA was obtained from cell pellets following the instructions of the Qiagen Genomic DNA Isolation kit (Valencia, CA, United States). The *VvcadA* gene (KTL38510) was obtained by PCR using the *V. vulnificus* genomic DNA as a template, and the following oligonucleotide primers: forward, 5′-CGG GGATCC ATG AAT ATT TTC GCT ATC TTG-3′ and reverse, 5′-CCC AAGCTT TCA GTC TTT CAG TAC TTT TAC-3′. The primers contained introduced *Bam*HI and *Hind*III restriction sites (underlined), respectively. The positive construct was sequenced at BGI (Shenzhen, China). *E. coli* BL21(DE3)/*pET28-VvcadA* was growth in LB broth until the OD_600_ reached 0.7, and then 1 mM isopropyl-β-D-thiogalactopyranoside (IPTG) was added. Cells were harvested after 4 h IPTG induction and were resuspended in lysis buffer. An imidazole gradient (50–250 mM) was used to elute VvCadA. Fractions containing the decarboxylase activity were pooled and dialyzed overnight against 50 mM MES buffer containing 100 mM NaCl (pH of the solution was 6.0). Sodium dodecyl sulfate polyacrylamide gel electrophoresis (SDS–PAGE, 12.5%) was used to separate purified VvCadA. Protein concentrations were determined using the Bradford method with bovine serum albumin as a standard (Bradford, 1976).

### Mutagenesis of VvCadA

The primers used for mutagenesis were: K367A, forward, 5′-ACA CAA TCC ACT CAC GCA CTG TTG GCG GCG TTC-3′; reverse, 5′-GAA CGC CGC CAA CAG TGC GTG AGT GGA TTG TGT-3′; E387A, forward, 5′-GGT GAA TTT GAC CGT GCA TCG TTC AAC GAA GCC-3′; reverse, 5′-GGC TTC GTT GAA CGA TGC ACG GTC AAA TTC ACC-3′; E391A, forward, 5′-CGT GAG TCG TTC AAC GCA GCC TTT ATG ATG CAC-3′, reverse, 5′-GTG CAT CAT AAA GGC TGC GTT GAA CGA CTC ACG-3′; D519A, forward, 5′-GTG GCG AAA TAT CTT GCA GAA CGT GGC ATT GTG-3′, reverse, 5′-CAC AAT GCC ACG TTC TGC AAG ATA TTT CGC CAC-3′. All the mutants were confirmed by DNA sequencing at BGI (Shenzhen, China). The mutant proteins (K367A, E387A, E391A, and D519A) were purified using the methods described above.

### Gel Filtration Chromatography

Gel filtration chromatography was achieved using a Sephacryl S-300 HR column (GE Healthcare) on a fast protein liquid chromatography (FPLC) system at the flow rate of 0.5 mL/min. The column was equilibrated with 50 mM MES/100 mM NaCl solution (pH 6.5). Protein standards included IgM (970 kDa), thyroglobulin (669 kDa), ferritin (440 kDa), catalase (232 kDa), and aldolase (140 kDa).

### Assays for VvCadA Activity

VvCadA (10 μg/mL) was mixed with 10 mM lysine in 50 mM MES buffer (pH 6.0) containing 1 nm PLP. After incubation for 20 min at 37°C, 1 ml of 1 M potassium carbonate was added to stop the reaction. One milliliter of 10 mM 2,4,6-trinitrobenzenesulfonic acid was added and samples were incubated for 5 min at 40°C, after which 2 ml toluene was added and samples were vortexed for 20 s. The absorbance of the toluene layers was read at 340 nm against a blank prepared from the toluene extracts where potassium carbonate was added before the enzyme. To check the temperature effect, assays were performed at a temperature gradient from 15 to 50°C. The effect of pH was checked following the methods described above.

The kinetic characters such as initial velocities, maximal velocity and *Michaelis* constants were analyzed by experiments with different lysine concentrations (0.1–2 mM) under the optimal conditions (pH 6.0, 37°C, in 50 mM MES solution). Sigma Plot software with a non-linear regression *Michaelis and Menten* model was used. Every point was determined by three separate experiments.

### Quantitative RT-PCR (RT-qPCR)

Culture of *V. vulnificus* was performed as described above. Bacterial cells in exponential phase were harvested and washed (PBS, pH 7.0, 0.85% NaCl). For low pH stress, the washed cells were incubated for 60 min (sodium acetate buffer, pH 5.0, 0.85% NaCl). For oxidative stress, the cells in exponential phase were shocked by addition of 5 mM H_2_O_2_ and incubation for 60 min. For the low-salinity-adapted culture, methods followed published literature (Wong and Liu, 2008). TRIzol reagent (Invitrogen, CA, United States), DNAase treatment (Thermo Scientific Fermentas, Germany) and the RiboClone cDNA Synthesis System (Promega, United States) were used to produce cDNA according to the manufacturers’ instructions. SYBR Green PCR Master Mix (Toyobo, Japan) was used in Real-time PCR. The reaction products were serially diluted. Expression of *VvcadA* was analyzed by using the following primers: 5′-TGT GGT GTG CTG TTT GAC TG-3′ and 5′-ACG TTT AGG CGT AGA TCG GT-3′ (with 94.8% amplification efficiency). Expression of *speF* was analyzed by using the following primers: 5′-GGG CGA AAT CTG GAC TCA AC-3′ and 5′-ACC TTG AAT CTC TGG TGC GA-3′ (with 96.0% amplification efficiency). Normalization was carried out with 16S rRNA (LOSI01000001) levels, which were amplified with primers 5′-AAT TCG ATG CAA CGC GAA GA-3′ and 5′-CGA CAT TAC TCG CTG GCA AA-3′ (98.2% amplification efficiency) The cDNA template was diluted 10-fold to 10^−3^, and qRT-PCR was performed to examine each primer efficiency. The primer efficiency was analyzed by calculating the equation *E* = 10^[1/slope]^ and primer sets with an *E*-value ranging from 1.91 to 2.05 were used in this study (De Filippis et al., 2013). The threshold cycle (C_T_) of each time point was compared to the zero time point to obtain ΔC_T_ (Ct other time point – Ct 0 min). Next, the gene of interest ΔC_T_ was compared to the reference gene ΔC_T_ to obtain the ΔΔC_T_ value (ΔC_T_ gene of interest – ΔC_T_ reference gene) for each time point. Expression of each gene of interest in 0 min was defined as 1. Three independent experimental and technical replicates were performed.

### Cadaverine Concentration Determination

Cadaverine concentration was determined in the crude extracts from the cultures of the different stressors mentioned above (12,000 *g* for 5 min at 4°C). When necessary, samples were diluted before analysis (De Filippis et al., 2013). Pre-column o-phthaldialdehyde derivatization coupled with reverse-phase HPLC, wavelength detector (G1314A; Agilent Technologies, Palo Alto, CA, United States) at 230 nm, were used to detect cadaverine concentration with standard cadaverine dihydrochloride (Tokyo Kasei Kogyo Co., Ltd.) (Qian et al., 2011).

### Protein Sequence Analysis

Protein molecular weight and pI were calculated on the ExPASy Server (Gasteiger et al., 2005). Multiple sequence alignments were carried out by the ClustalOmega program (Sievers et al., 2011). Phylogenetic trees were reconstructed by MEGA 7.0 using the maximum-likelihood method and 1000 iterations (Kumar et al., 2016).

### Construction of Sequence Similarity Network (SSN)

InterPro webpage^1^, sequences were used to download sequence and analyze the ornithine/lysine/arginine decarboxylases in global form (Mitchell et al., 2015). Enzyme Function Initiative-Enzyme Similarity Tool (EFI-EST) was used to make the SSNs (Gerlt et al., 2015). The results were visualized by Cytoscape 3.3 (Shannon et al., 2003) using 90% protein identity and cutoff *e*-value to display node and edge, respectively.

### Statistical Analysis

Mean, standard deviation (SD), and analysis of variance (ANOVA) were calculated using EXCEL software. Statistically significant differences were determined by ANOVA.

## Results

### Cloning, Expression, and Purification of VvCadA

**FIGURE 1:**
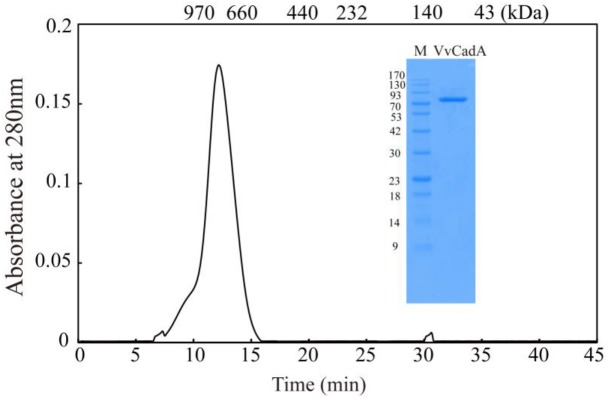
Purification and gel filtration chromatography of VvCadA. Purifed VvCadA was analyzed by Sephacryl S-300 column with a flow rate of 0.5 mL/min. The insert shows the proteins that were electrophoresed on a 12.5% SDS–PAGE and stained with Coomassie brilliant blue G-250. Lane M, protein marker; the molecular mass standards are indicated on the left.

The *VvcadA* ORF contains 2136 nucleotides coding for a protein of 711 amino acids (aa), with a theoretical pI of 5.53 and MW of 80.43^2^. This *VvcadA* gene was obtained from *V. vulnificus* genomic DNA through PCR. The recombinant VvCadA containing an N-terminus 6 × His tag was expressed from *E. coli* after 1 mM IPTG induction (Figure 1). The purified protein was visualized as an 82-kDa protein using SDS–PAGE (Figure 1). According to the gel filtration chromatography analysis, VvCadA is a decamer (∼800 kDa) composed of 82 kDa subunits.

### Optimal Temperature and pH for Activity of VvCadA

**FIGURE 2:**
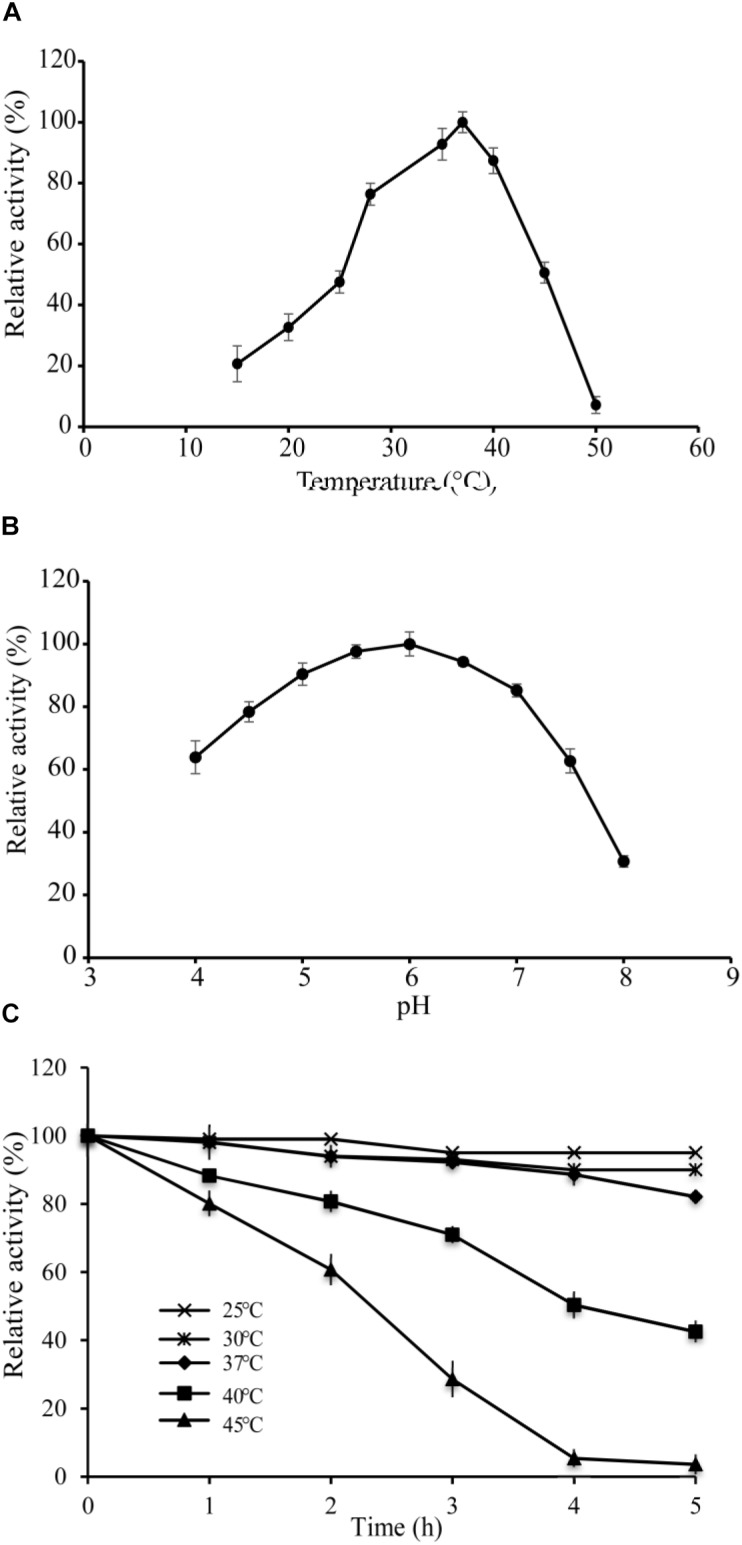
Enzyme activity assays of VvCadA. **(A)** Optimal temperature of VvCadA activity. **(B)** Optimal pH of VvCadA activity. Different buffers were used for the different pH solutions used in this assay. sodium acetate buffer was used for pH 4.0–4.5; MES buffer was used for pH 5.0 and 7.5; HEPES buffer was used for pH 8.0. **(C)** Effect of temperature on the stability of recombinant VvCadA. The purified enzyme was pre-incubated at 37°C (cycle), 40°C (diamond), and 45°C (triangle) for different times and the residual activities of the enzymes were measured.

The optimal temperature of VvCadA was determined by measuring decarboxylase activity of VvCadA from 15 to 50°C at a constant pH of 6.0. The recombinant enzyme was active above 15°C, and its activity increased with temperature elevation (Figure 2A). The optimum temperature for VvCadA was around 37°C. To understand the role of pH, decarboxylase activity of the purified enzyme was investigated between pH 4.0 and 8.0 at a constant temperature of 37°C. VvCadA was most active between pH 5.0 and pH 7.0 (Figure 2B). Relative decarboxylase activity of VvCadA increased with increasing pH, peaking at pH 6.0, then decreased to 60% at pH 7.5.

### Thermostability of VvCadA

To determine the thermostability of VvCadA, samples of the purified enzyme were incubated at 25, 30, 37, 40, and 45°C at pH 6.0 in MES buffer. As shown in Figure 2C, with the increase in temperature, the relative activity gradually reduced, and also with the extension of time, the relative activity gradually decreased. The relative decarboxylase activity of VvCadA at 37°C remained at 90% after 2 h and 80% after 4 h. The enzyme lost activity at high temperature, for example, the relative activity reduced to 30% after 3 h at 45°C. However, the enzyme was quite stable at 25 and 30°C; activity remained >90% after 4 h at these temperatures.

**FIGURE 3:**
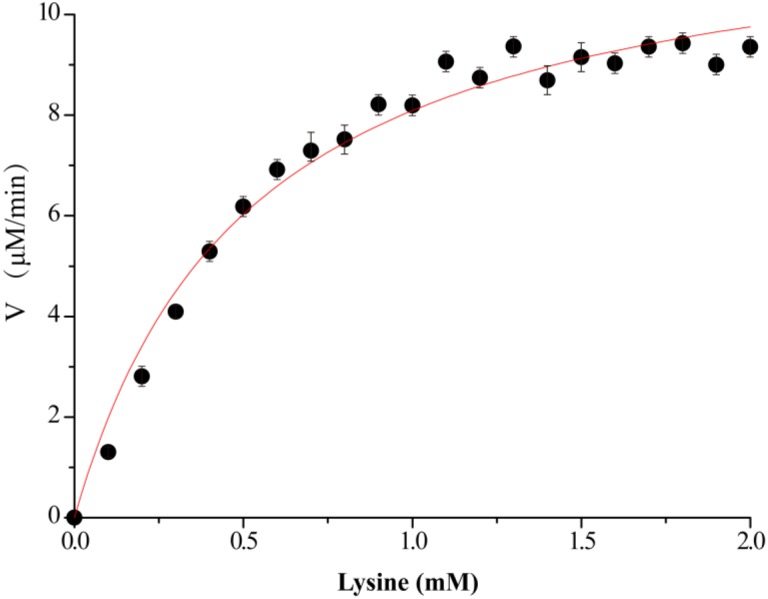
Kinetics assay of VvCadA. The velocity data changed with the increase of substrate concentrations were fitted to the *Michaelis–Menten* equation by non-linear regression calculations.

**FIGURE 4:**
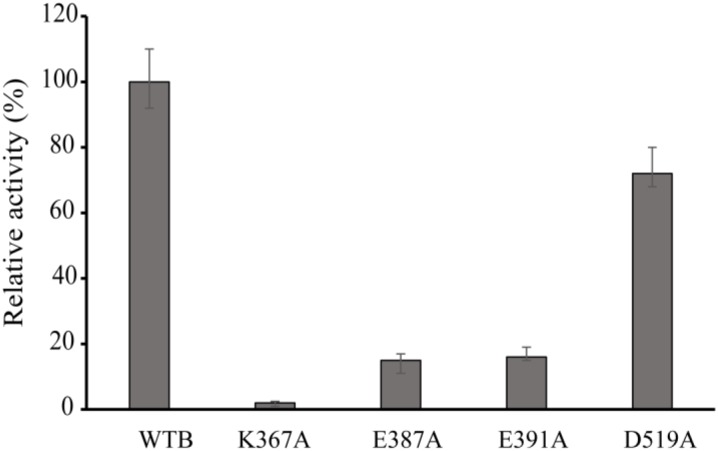
Relative activity of wild-type VvCadA, VvCadA-K367A, VvCadA-E387A, VvCadA-E391A, and VvCadA-D519A. All experiments were performed in triplicate. The error bars mean the standard deviation (SD) of three measurements.

### Enzyme Kinetics

The kinetics of recombinant VvCadA were analyzed using different concentrations of lysine as a substrate (Figure 3). The reaction was performed in a MES buffer (pH 6.0) at 37°C with lysine concentrations ranging from 0.1 to 2.0 mM. The *K*_m_ and *V*_max_ values for VvCadA were calculated using the data obtained with different substrate concentrations. VvCadA could catalyze lysine decarboxylation with an apparent *K_m_* of 0.45 ± 0.05 mM, *V*_max_ of 9.45 ± 0.24 μM/min, and *k*_cat_ of 1.58 ± 0.04/sec (*n* = 9).

**FIGURE 5:**
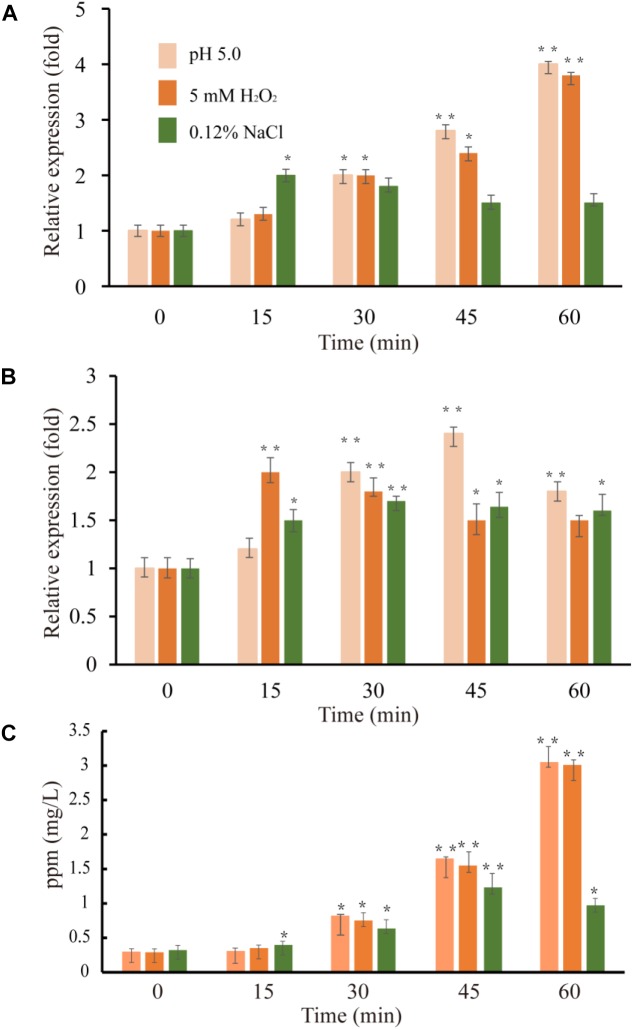
Expression level of *VvcadA* **(A)** and *VvspeE* **(B)** genes and cadaverine concentrations in the supernatants **(C)** under low pH (pink), oxidative (orange), and low-salinity (green) stresses. The culture and treatment of *V. vulnificus* were described as in “Materials and Methods.” The mRNA relative quantity of *VvcadA* from *V. vulnificus* cells treated by different stresses was measured by RT-qPCR and indicated as fold difference from the value of the untreated cells, which is taken as 1. The concentration of cadaverine under different stresses was measured as described in “Materials and Methods.” Error bars indicate the SDs from three independent experiments. ^∗^*p* < 0.05 and ^∗∗^*p* < 0.01 compared with the values under normal conditions.

### Effects of Point Mutation on Enzymatic Activity

Multiple sequence alignments indicated that VvCadA had 75 and 67% sequence identity to inducible lysine decarboxylase and constitutive lysine decarboxylase of *E. coli*, respectively (Supplementary Figure 1). The results further revealed the Lys367 (K367) residue, which is responsible for the PLP binding (Kanjee et al., 2011a), was highly conserved. Several negatively charged residues including E387, E391, and D519 that may interact with the positively charged substrates were also conserved based on the analysis (Supplementary Figure 1). To confirm the function of the important amino acids, residues K367, E387, E391, and D519 were mutated. The mutant recombinant proteins were expressed and purified, and showed a similar elution pattern in gel chromatography as the wild-type protein (Supplementary Figure 2), suggesting that mutagenesis does not affect oligomerization. The activities of these mutant proteins were measured together with the wild-type enzyme (Figure 4). The protein with a mutation in the residue involved in PLP binding (K367A) had no detectable decarboxylase activity. The activities toward lysine by the point mutants of E387A and E391A at the substrate binding sites was approximately 15% of that of wild-type VvCadA, however, the D519A mutant had 70% of the activity of the wild-type enzyme. These experimental findings confirmed that the substrate binding site near the PLP binding site is also important for catalyzing lysine decarboxylation.

**FIGURE 6:**
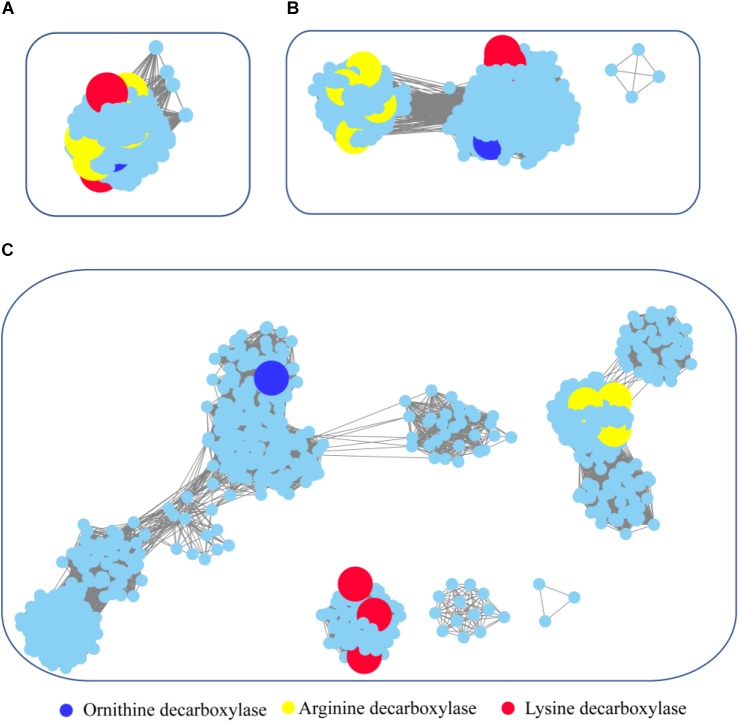
Global view of sequence relationships in the PLP-dependent aminotransferases superfamily. PLP-dependent aminotransferases in the Interpro database were analyzed by sequence similarity network (SSN) with an *e*-value threshold of 10^−20^**(A)**, 10^−25^**(B)**, and 10^−30^**(C)**. The network displayed 927 nodes representing 6,746 proteins filtered at 90% sequence identity. Nodes annotated by Swiss-Prot are enlarged. Annotated nodes with different colors a are listed on the bottom. The proteins that are not studied experimentally are shown in cyan color.

### Expression Profile of the Genes VvcadA and VvspeF Under Stress Conditions

Previous studies showed that lysine decarboxylase in *V. vulnificus* was up-regulated in the presence of low pH and secondary oxidative stress (Kim et al., 2006). The inducible ornithine decarboxylase, SpeF, also plays an important role of for acid stress resistance in bacteria (Del Rio et al., 2018). We used qRT-PCR to compare the expression of the genes *VvcadA* and *VvspeF* in response to different abiotic stresses. The relative expression patterns of *VvcadA* and *VvspeF* are shown in Figure 5. Under low pH and oxidative treatment, the transcript levels of *VvcadA* and *VvspeF* showed obvious increases. The transcript level of *VvcadA* increased fourfold after 1 h treatment of low pH and oxidative stress, while the expression level of *VvspeF* increased less than twofold. In natural and food-processing environments, pathogenic bacteria face several common stressors including low salinity (Wong and Liu, 2008). During low-salinity treatment, the expression levels of the two genes increased in a similar pattern, showing approximately a twofold increase. These results suggested that the two decarboxylases may play important roles in the defense against various stresses, and that *VvcadA* may be more important during the process. Furthermore, we measured the concentrations of cadaverine under the stresses. The concentration of cadaverine under low pH and oxidative stress increased around 10-fold after 1 h of treatment. Under low salinity conditions, the concentration of cadaverine increased around three-fold. This further indicated that VvCadA and its product play important roles in stress tolerance.

### A Global View of Evolutionary Relationships in the PLP-Dependent Aminotransferase Superfamily

For maintenance of intracellular pH, organisms have developed diverse mechanisms including amino acid decarboxylation reactions. We analyzed the genome sequences of serval *Vibrio* strains, and the genes encoding carboxyspermidine decarboxylase, diaminopimelate decarboxylase, ornithine decarboxylase, and lysine decarboxylase were discovered. Among these genes, ornithine decarboxylase (SpeE) and lysine decarboxylase have been reported to be involved in acid tolerance (Azcarate-Peril et al., 2004). The maximum-likelihood phylogenetic tree for ornithine decarboxylase and lysine decarboxylase from *Vibrio*, *E. coli*, *Selenomonas ruminantium*, and *Salmonella typhimurium* is presented in Supplementary Figure 3. The ornithine decarboxylases from *Vibrio metoecus*, *Vibrio albensis*, and *Vibrio cholerae* were in the same cluster with constitutive and inducible ornithine decarboxylase from *E. coli* (Kanjee et al., 2011b). Meanwhile, the ornithine decarboxylases from *Vibrio natriegens*, *Vibrio vulnificus*, *Vibrio fluvialis*, and *Vibrio furnissii* were localized together with lysine/ornithine decarboxylase from *Selenomonas ruminantium*, which has decarboxylase activities toward both lysine and ornithine (Takatsuka et al., 2000). However, genes encoding lysine decarboxylase showed a much more conserved distribution in *Vibrio* strains.

To survey detailed sequence similarity across the superfamily, a SSN for 6746 sequences of ornithine/lysine/arginine decarboxylases in the InterPro database was constructed (*e*-value cutoff of 10^−20^, sequence identity >30%) (Figure 6; Jia et al., 2017). Almost all the proteins were located in one cluster, and the lysine decarboxylases, ornithine decarboxylases, and arginine decarboxylases were included in the cluster. Arginine decarboxylases can be separated from lysine decarboxylases with ornithine decarboxylases, since the threshold stringency is lower to *e*-value 10^−25^ (sequence identity >∼40%). When the network was displayed at the value of 10^−30^ (sequence identity >50%), the three decarboxylases were classified into different clusters and experimentally verified enzymes were identified in each group. This analysis indicated that PLP-dependent aminotransferases are highly conserved even though they showed different substrate specificity.

To explore the occurrence of PLP-dependent aminotransferases in bacteria, taxonomic classification was performed in the network (Supplementary Figure 4). The relative abundance of the proteins is widely diverse among the taxonomic classes. In archaea, members of PLP-dependent aminotransferases were found only in the class of Methanomicrobia. The prevalence of PLP-dependent aminotransferase genes in bacteria was high in Gammaproteobacteria, Betaproteobacteria, and Alphaproteobacteria. Actinobacteria and Bacilli also showed relatively high abundance. Interestingly, the clusters with ornithine decarboxylase and arginine decarboxylase contained the proteins belonging to members of different classes, while the cluster with lysine decarboxylase contained the proteins only from Gammaproteobacteria. This suggested that horizontal gene transfer occurred during the evolution and distribution of ornithine decarboxylase and arginine decarboxylase, but this seldom happened for lysine decarboxylase.

## Discussion

There are two types of bacterial amino acid decarboxylase, inducible and constitutive. The former type includes decarboxylases for lysine, ornithine, arginine, S-adenosyl-L-methionine, and diaminopimelic acid (Kikuchi et al., 1997). In bacteria and plants, two different genes code for lysine decarboxylase. One, *cadA*, is induced by changes in external pH, increased lysine and low concentration of oxygen. The other gene, *ldcC*, is constitutively expressed, and is independent of pH changes (Krithika et al., 2011). *V. vulnificus* is highly lethal as it is responsible for the majority of reported cases of seafood-related deaths. In this study, we cloned, expressed, and biochemically characterized a lysine decarboxylase from *V. vulnificus* (VvCadA) for the first time. The recombinant enzyme exists as a decamer with a native molecular mass of ≈800 kDa. Measuring the decarboxylase activity of the wild-type and various mutant recombinant VvCadA proteins revealed that the residues binding LPL and lysine are critical for the activity. Furthermore, the transcription level of *VvcadA*, that was higher than that of *VvspeE*, was up-regulated by low pH, oxidative, and low salinity stresses. The concentrations of cadaverine were also increased under these conditions. Considering the importance of VvCadA to protect *V. vulnificus*, we propose that targeting VvCadA could be of benefit for controlling the growth of *V. vulnificus*.

The previously determined structure of CadA from *E. coli* showed that the protein is an oligomer of five dimers which form a decamer (Kanjee et al., 2011a). Sequence alignments revealed that VvCadA showed high identity to CadA from *E. coli*. In addition, gel filtration chromatography proved that VvCadA also forms a decamer. Bioinformatics analysis indicated that only one gene encoding lysine decarboxylase occurs in the genome of *V. vulnificus*, while *E. coli* harbors both genes (*cadA* and *ldcC*). This analysis suggested that CadA may have multiple functions in *V. vulnificus*. Previous studies also showed that *cadA* in *Vibrio cholerae* was induced during infections, and *in vitro* under conditions of low pH and high lysine concentration (Merrell and Camilli, 1999). Expression of *cadA* in *V. vulnificus* was induced by SoxR in superoxide stress (Kim et al., 2006). Our experiment confirmed the findings of previous studies and further showed that *VvcadA* can be induced by low salinity stress; the distribution of *V. vulnificus* in the environment is positively correlated with salinity (20–25 ppm) (Randa et al., 2004). Based on this evidence, we proposed that VvcadA plays diverse roles in the defense response of *V. vulnificus*.

pyridoxal-5′-phosphate-dependent enzymes were divided into α, β, and γ classes, but the functional classification did not always follow the evolutionary history (Alexander et al., 1994). In the current study, we performed a global analysis of ornithine/lysine/arginine decarboxylases which suggested these proteins shared a common ancestor and that ornithine/lysine decarboxylases are more closed in evolution. Horizontal gene transfer of lysine decarboxylase seldom occurs compared with the other two proteins. Furthermore, the lysine decarboxylases formed a compact cluster compared with ornithine/arginine decarboxylases, suggesting that lysine decarboxylases are highly conserved during evolution.

The product of decarboxylation of lysine is cadaverine, which has immense applications in drug development and other industries. Limited works showed direct cadaverine production from lysine, although many development in the technology of fermentation biotransformation (Kim et al., 2015). The direct conversion of lysine to cadaverine based on the engineered *E. coli* has been proved to be a feasible and practical approach (Kim et al., 2015). Considering that VvCadA has similar catalytic properties as CadA from *E. coli* (Qian et al., 2011), our results provide another choice that is suitable for industrial applications.

## Author Contributions

XH, HY, and LH designed the project and analyzed the data. XH contributed to bioinformatic analysis. LH, JY, XA, and SL contributed to molecular biology studies. XH, HY, and LH wrote the manuscript. All authors reviewed and edited the manuscript.

## Conflict of Interest Statement

The authors declare that the research was conducted in the absence of any commercial or financial relationships that could be construed as a potential conflict of interest.

## Footnotes

1 http://www.ebi.ac.uk/interpro/entry/IPR011193,68.0

2 http://web.expasy.org/compute_pi/
