# Supplementary material for: Biochemical Characterization and Phylogenetic Analysis of the Virulence Factor Lysine Decarboxylase From Vibrio vulnificus
Source: Front Microbiol. 2018 Dec 11;9:3082. doi: 10.3389/fmicb.2018.03082 (PMC6297170; doi:10.3389/fmicb.2018.03082)
Supplement: Supplementary file 1 [file Data_Sheet_1.docx]

**Biochemical Characterization and phylogenetic analysis of a Lysine Decarboxylase, a Virulence Factor of *Vibrio vulnificus***

Supplementary Fig. 1

**
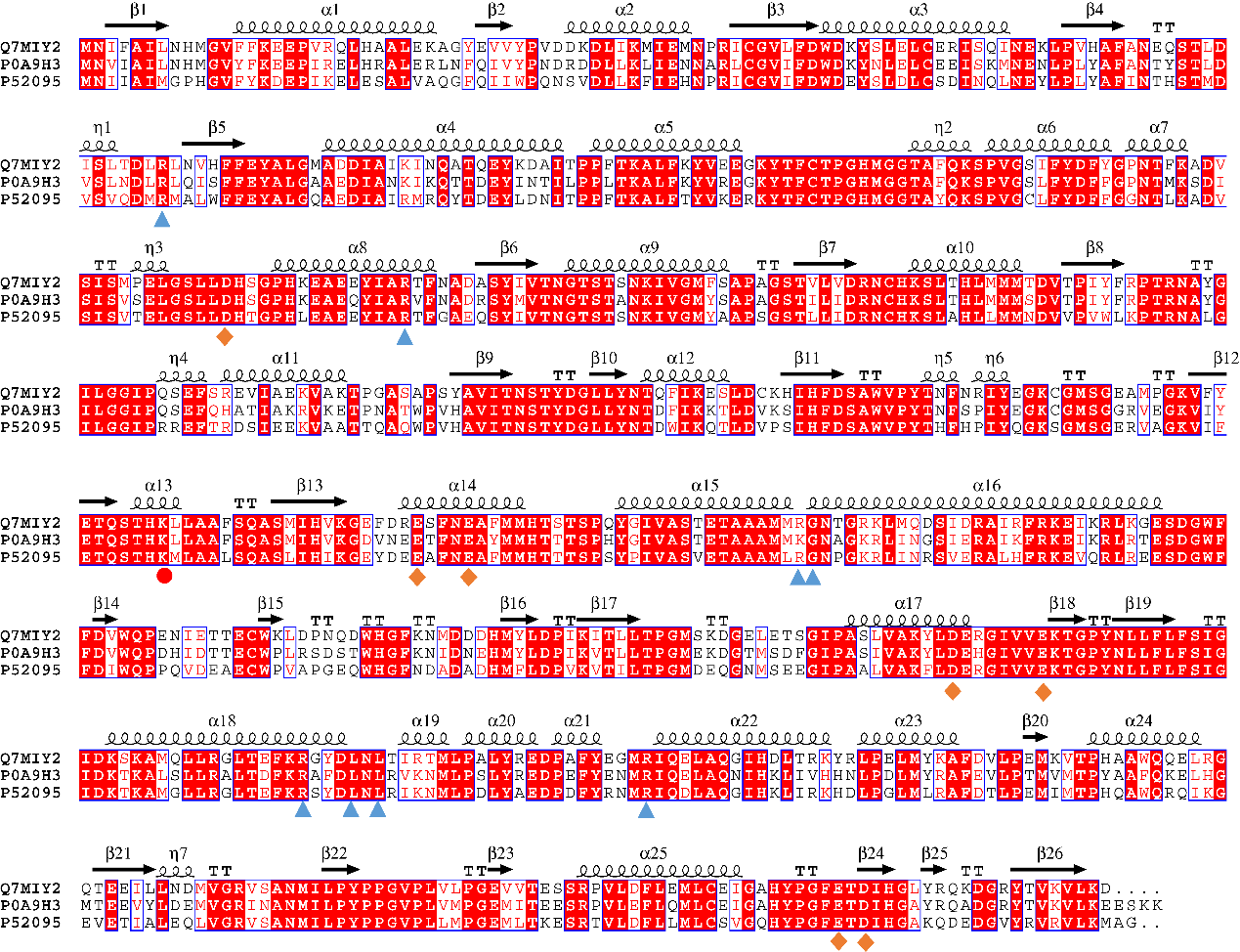
**

Supplementary Fig. 1. Sequence alignment of VvCadA and other homologs. The residues involved in PLP binding are indicated by red circle. The amino acid residues involved in the substrate binding are highlighted by orange diamonds and the residues binding ppGpp are highlighted by blue triangles. The predicted secondary structure is shown at the top of the alignment. α-helices are represented as helices, β-strands are represented as arrows, β-turns are represented as “TT,” and 310-helices are represented as η.

Supplementary Fig. 2


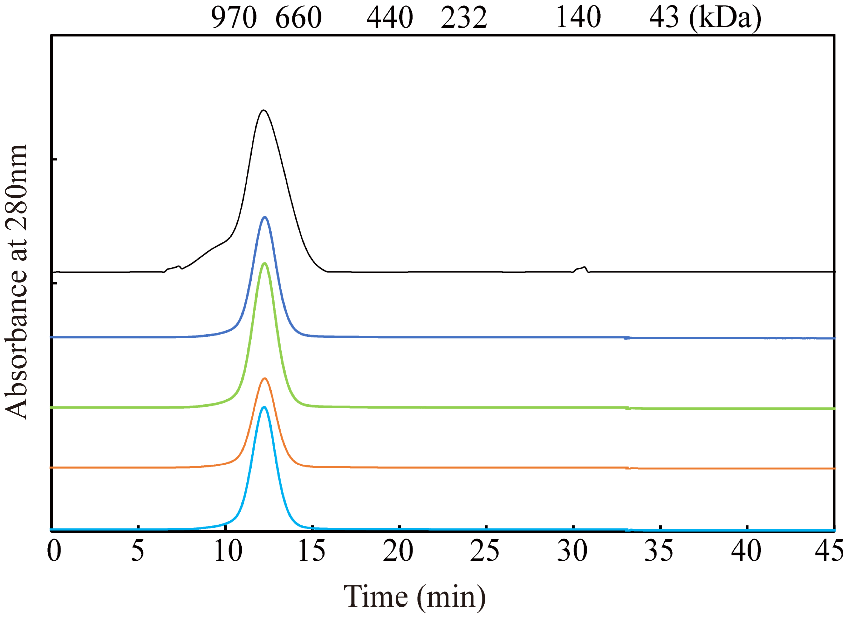


Supplementary Fig. 2. Gel filtration chromatography of wide type VvCadA (black) and the mutants of K367 (blue), E387 (green), E391 (orange), and D519 (light blue).

Supplementary Fig. 3


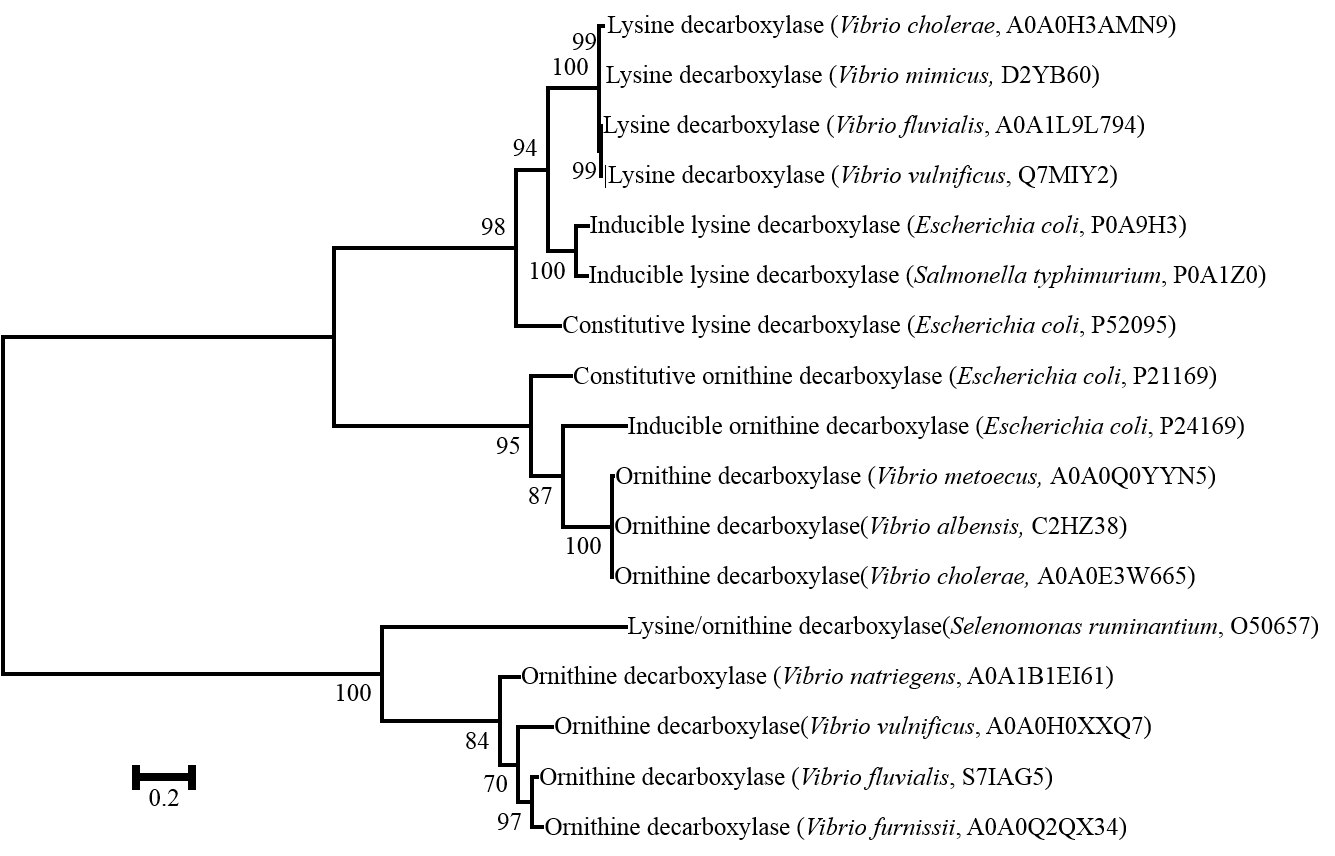


Supplementary Fig. 3. Phylogenetic and sequence analysis of VvCadA and other homologs. (A) Molecular phylogenetic analysis of lysine decarboxylases by maximum likelihood method generated using MEGA7. The evolutionary history was inferred by using the maximum likelihood method based on the JTT matrix-based model. The percentage of replicate trees in which the associated taxa clustered together in the bootstrap test (1000 replicates) is shown next to the branches. The accession numbers of the proteins are indicated after the names’ of the species.

Supplementary Fig. 4

**
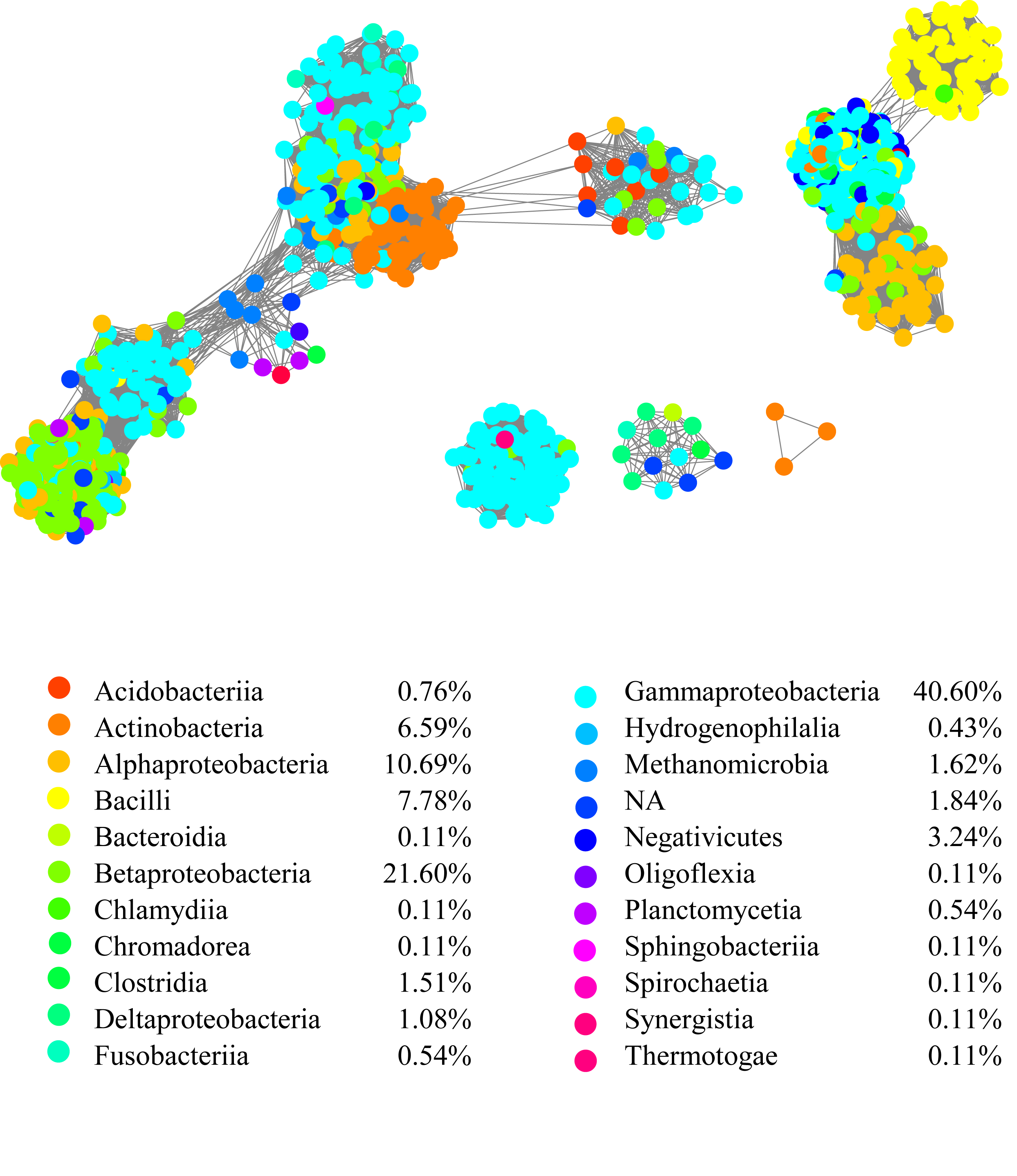
**

Supplementary Fig. 4. Taxonomic (class) distribution of PLP-dependent aminotransferases in prokaryotes. Nodes from the same taxonomic groups in the global network are the same color. The color corresponding to each phylum and protein percentage in each phylum are listed on the bottom.
